# NAG-PEGylated multilamellar liposomes for BBB-GLUT transporter targeting

**DOI:** 10.1080/2331205x.2019.1701343

**Published:** 2020-01-08

**Authors:** Nahid S. Kamal, Muhammad J. Habib, Ahmed S. Zidan, Pradeep K. Karla

**Affiliations:** 1 Department of Pharmaceutical Sciences, Howard University, 2300 4^th^ Street NW, Washington, DC 20059, USA; 2 Arnold & Marie Schwartz College of Pharmacy & Health Science, Long Island University, Brooklyn, NY, USA; 3 Department of Pharmaceutics and Industrial Pharmacy, Faculty of Pharmacy, Zagazig University, Zagazig, Egypt

**Keywords:** Pharmaceutical Science, Analysis & Pharmaceutical Quality, Pharmacy, Biopharmaceutics, Drug Discovery, Drug Design & Development, N-acetyl Glucosamine (NAG), Glucose (GLUT) Transporter, Blood-Brain Barrier (BBB), transporter targeted drug delivery, liposomes

## Abstract

The primary objective of the research study is to investigate Glucose (GLUT) transporter targeting of the drug (Citalopram-Hbr) for increased permeability across the Blood-Brain Barrier (BBB). The current study reports the development, physicochemical characterization, cytotoxicity analysis and in-vitro BBB permeability assessment of the Citalopram-Hbr liposomal formulations. Rat Primary Brain Microvascular Endothelial Cells (RPBECs) were used for cytotoxicity analysis and drug permeability testing. Five N-Acetyl Glucosamine (NAG) coated PEGylated multilamellar liposomal formulations were prepared and tested. Permeability of the liposomal formulations was evaluated in RPBECs monolayer. The particle size of the formulations ranged from 13 to 4259 nm. Entrapment efficiency was 50–75%. Cytotoxicity analysis indicated viability (>90%) for all five formulations (0.3–1.25 mg/ml). Apparent drug permeability (Papp) of the formulations ranged from 5.01 × 10^4^ to 15 × 10^4^ cm/min. The study demonstrated successful preparation of NAG-coated PEGylated multilamellar liposomal formulations with high drug entrapment efficiency. Cytotoxicity data indicated that the formulations were well tolerated by the cells up to a concentration of 1.25 mg/ml. Transport study data demonstrated that RPBMECs monolayers can be employed as a robust screening tool for future drug transport studies targeting GLUT transporter on the BBB. The drug permeability values provide a promising preliminarily proof that NAG-coated liposomal formulations can be an effective tool for BBB-GLUT transporter targeting.

## 1. Introduction

BBB is a complex structure of endothelial cells and tight junctions restricting the permeability of a wide array of drug molecules (Pardridge & Oldendorf, 1977). Several carrier-mediated transport systems that aid in the transport of the nutrients and endogenous molecules were identified on BBB (Borlongan & Emerich, 2003). The major BBB endogenous transporter systems include Glucose transporter (GLUT), Mono Carboxylic Acid transporter (MCT), Neutral Amino Acid and Amine transporter system (SLC). These transporter systems were found to have varied ability to transport the endogenous molecules (Erdlenbruch et al., 2003). The transporter capacity of the GLUT transporter was found to be significantly higher than other transporter systems expressed on the BBB (GLUT > MCT >SLC) (Begley, 2004; Pardridge, 1983). Various homologs of GLUT transporter system include GLUT1, GLUT2, GLUT3, GLUT4, GLUT5, GLUT6, and GLUT7 (Maher, Vannucci, & Simpson, 1993; Pardridge, Farrell, & Farrell, 1990). Among them, GLUT1 was found to be widely expressed on the BBB endothelium and constitute >90% of all the GLUT transporters (Huang, Shen, & Wu, 2009; Pardridge, 1983)

Various formulation strategies were explored with emphasis on endogenous transporter targeting to increase the permeability of drugs across the BBB. For example, coumarin six-loaded liposomal formulations with glucose residuals demonstrated increased permeability across the BBB due to GLUT transporter targeting (Gould & Holman, 1993). Another study reported that intravenous administration of glucose-coated niosome formulations of Vasoactive Intestinal Peptide (VIP) resulted in increased peptide permeability across the BBB and increased concentrations of VIP in selective brain tissues of the mice (Pardridge, 2007). Further, Qin et al. reported that liposomal formulations containing glycosyl derivatives with coumarin-6 as a fluorescence probe demonstrated less cytotoxicity than the conventional liposomal formulations (QIN et al., 2010). These studies established GLUT transporter system as a promising drug delivery target for increased permeability of drugs across the BBB.

The main objective of this research study is to investigate GLUT transporter targeting of the Citalopram-Hbr for increased permeability across BBB. The study demonstrates the development of pegylated multilamellar liposomal formulations of Citalopram-Hbr with N-acetyl glucosamine (NAG) as a glucose derivative ligand for selective GLUT transporter targeting. In addition, the study reports a comprehensive physicochemical characterization, cytotoxicity analysis and in-vitro BBB permeability assessment of the Citalopram-Hbr liposomal formulations.

## 2. Materials and methods

### 2.1. Materials

Hydrogenated phosphatidylcholine (95%-hydrogenated phosphatidylcholine and 0.5%hydrogenated lyso phosphatidylcholine) was gifted from American Lecithin Company (Oxford, CT). Cit-HBr was kindly donated by Lund beck A/S (Copenhagen, Denmark). Diacetyl phosphate, NAG, and cholesterol were purchased from Fisher Scientific (Pittsburgh, PA). PEG 600 was purchased from Croda Inc. (Columbus Circle Edison, NJ). All other solvents were purchased as HPLC grade. Rat Primary Brain Microvascular Endothelial Cells (RPBMECs), Animal Endothelial cell medium supplemented with murine Endothelial Growth Factor (mEGF), fetal bovine serum (FBS), L-glutamine, Antibiotic-Antimycotic and gelatin-based coating solutions were purchased from Cell Biologics (Chicago, IL). Hank’s Buffered Salt Solution (HBSS) was purchased from Thermo Scientific (South Logan, UT). CellTox Green Cytotoxicity Assay kit was gifted by Promega Corporation (Madison, WI). D-Luciferin fluorescence dye was purchased from BD Bioscience (Bedford, MA). Trypsin was purchased from Gibco (Grand Island, NY). HEPES buffer was purchased from MP Biomedical (Solon, OH). Polystyrene nonpyrogenic 96-well plates were obtained from BD Bioscience (Bedford,MA). 24-well trans inserts obtained from Corning Life Sciences (Lowell, MA.).

### 2.2. Methods

#### 2.2.1. Preparation of n-acetyl glucosamine coated pegylated multilamellar liposomal formulations

Five formulations of N acetyl glucosamine (NAG) coated pegylated multilamellar liposomal formulations were prepared by incorporating different concentrations of phospholipids, cholesterol, diacetyl phosphate, NAG, and polyethylene glycol (PEG 600) in chloroform as showed in Table 1. The liposomes were prepared by using the thin-film method as described by Evjen, Hagtvet, Nilssen, Brandl, and Fossheim (2011). The preparation method involved dissolving all the lipids in the organic solvent, forming dry film after solvent evaporation, and finally, the dry film was hydrated by using various concentrations and volumes of isotonic Cit-HBr drug solutions. The liposomal suspension was exposed to the different physio-chemical characterization namely entrapment efficiency, particle sizing, zeta potential, polydispersity and in vitro release by using the methods described previously in detail (Kamal, Cutie, Habib, & Zidan, 2015). The free drug from the liposomal suspension was removed by ultracentrifugation (Beckman XL-90 ultracentrifuge, Beckman, Fullerton, CA) of 65 000 rpm for 2 hours at 4°C. The resultant liposomal residue was stored at 20°C for further analysis after reconstituted with 5 mL of isotonic solutions.

#### 2.2.2. Cell culture

Rat Primary Brain Microvascular Endothelial Cells (RPBMECs) (Cell Biologics, Catalog No. RN 6023) were isolated from brain tissue of 1-day-old neonatal laboratory Sprague-Dawley rats. Cells were grown in T75 tissue culture flasks and incubated generally for 3–7 days. Frozen cryo-preserved cells in vials (1×10^6^ cells per ml) at passage three were shipped. Cells were stored in the liquid nitrogen (−180°C) until ready for experimental use. Prior use cells were thawed at room temperature and were grown as per the protocol supplied by the vendor (CellBiologics). Briefly, the cells were plated in a 75 cm^2^ culture flask pre-coated with gelatin-based coating solution. The plated cells were maintained in an incubator at 37°C, 5% CO_2_, and 95% relative humidity. The cells were allowed to adhere and the media were replaced every 24 h. At the desired confluence (~ 80%), the cells were passaged and sub-cultured as per supplier protocol. Passage numbers ranging from 4 to 8 were used for cytotoxicity analysis and drug permeability studies.

#### 2.2.3. Evaluation of cytotoxicity

Cytotoxicity assay was performed with the CellTox Green™ dye Cytoxicity test kit (Promega Corporation, WI, USA). The assay protocol provided by the test kit manufacturer was adapted with no further modifications. The kit employed proprietary asymmetric cyanine dye that favorably stained the DNA of dead cells compared to viable cells. The fluorescence measured from the stain of dead cells was proportional to cell toxicity. The cells were seeded in Polystyrene nonpyrogenic 96-well plate at a density of 10^4^ cells/well and were incubated at 37°C, 5% CO_2_, 95% relative humidity, and allowed 24 h to attach. Test formulations were added to the wells (n = 4) at four concentrations (0.312, 0.625, 1.25, 2.5 mg/ml). For positive control wells (lysis solution provided by the manufacturer), negative control (untreated cells) and blank (cultured medium) were employed. After treatment with liposomal formulations, the cells were incubated for 24 h and the fluorescence was measured at excitation (485–500 nm) and an emission (520–530nm) wavelengths utilizing CytoFluor Series 4000 Fluorescence Multi-Well Plate Reader (Applied Biosystems, Ca, USA). The cytotoxicity analysis was determined by plotting the percentage of cell viability vs. fluorescence.

## 3. In-vitro drug transport study

### 3.1. Cell culture in transwell inserts and monolayer integrity testing

The transport study was adapted from the published protocol by Vernon et al. with no further modifications (Vernon, Clark, & Bressler, 2011). Briefly, the 24-well transwell inserts were coated with gelatin-coating solution and allowed to dry for 30 min. The cells are plated in the transwells at a seeding density of ~4000 cells/cm^2^ and cultured in the incubator (37°C, 5% CO_2_, 95% relative humidity). Culture media were replaced every 72 h until the cells reached confluence and a clear monolayer was observed.

The monolayer integrity was studied by measuring the Trans Epithelial Electrical Resistance (TEER) and Lucifer Yellow (LY) paracellular permeability. The TEER of RPBMEC monolayers were equilibrated with the transport buffer (HBSS with 20mM HEPES, P^H^ 7.4). TEER was measured with an EVOM2 Epithelial Volt-Ohm Meter (WPI Inc., Sarasota, FL). The monolayers with TEER values ≥200 Ωcm^2^ and LY flux rates ≥1% were utilized for the transport studies.

The integrity of the cell monolayer was studied by determining the percent leakage of LY based on the earlier published protocol, with no further modifications (Dufes et al., 2004). The acceptable flux range of LY for intact cells was 0.3–2.0%. A flux value greater than 2% indicated the loss of monolayer integrity (QIN et al., 2010). The LY permeability assay was performed at the beginning and at the end of the transport study to ensure the integrity of the model was maintained.

After measuring the TEER, trans-well plates were washed thrice with transport buffer. One hundred microliters of LY solution (100μg/mL) was added to the donor well (apical) and 600 μL of HBSS was added to recipient well (basolateral). The transwells were incubated for 2 h at 37 °C and the apical to basolateral transport of LY was estimated. Fifty-microliter aliquots of transport buffer were collected from the basolateral chamber after a 2-h incubation. The collected samples were transferred to a 96-well plate and fluorescence intensity was measured at excitation (485 nm) and emission (535nm) wavelengths with CytoFluor Series 4000 Fluorescence Multi-Well Plate Reader (Applied Biosystems, MA USA).

The extent of “leakage” was calculated from the following equation (QIN et al., 2010):

%LYFlux=100×((LY−BL×Vol−BL)/(LY−AP×Vol−AP))

Where: LY-BL is the concentration of lucifer yellow in the basolateral receiver chamber [μM]

Vol-BL is the volume in the basolateral receiver chamber [cm^3^]

LY-AP is the concentration of lucifer yellow added to the apical donor chamber [μM]

Vol-APL is the volume in the apical donor chamber [cm^3^]

### 3.2. Drug permeability using sink condition

AP-BL transport of five liposomal formulations was evaluated across the RPBECs. The transport study was adapted from a published protocol by Vernon et al. (2011), with no further modifications. The pre-transport and post-transport monolayer integrities were evaluated by TEER measurements and LY leakage transport data analysis. Prior to the transport study, AP and BL sides of trans wells were washed thrice with transport buffer. The test compounds dissolved in transport buffer were added to the donor inserts (AP) at a concentration of 2.5 mg/ml (n = 4). Drug permeability was analyzed for 3 h and 100 μl buffer samples were withdrawn from the receiver chamber (BL) at predefined time intervals (15, 30, 60, 90, 120, 150 and180 minutes). The receiver chamber was replenished with 100 μl of transport buffer at each sample interval to maintain the sink conditions.

### 3.3. Drug transport sample analysis

The samples were analyzed in triplicate by High-Performance Liquid Chromatography (HPLC). The Shimadzu HPLC system was equipped with 2 LC-10ADvp pumps, DGU-14A degasser, SPD-10ADvp UV-Vis detector, SIL-10ADvp autosampler, and SCL-10Avp system controller. Mobile phase consisted of 25 mM KH_2_PO_4_ (pH = 3.9) and acetonitrile at 45:55 v/v ratio. Isocratic elution run was carried out on a Phenomenex Luna C18 column (250 mm × 4.6 mm, 5 um) at a flow rate of 0.8 ml/ min. The sample analysis wavelength was fixed at 240 nm and injection volume was 10 μL. The steady-state flux (J) of the drug across the cell monolayer was obtained from the slope of cumulative amount of drug transported vs. time plots by employing equation 1. The apparent permeability of the five formulations is estimated by equation 2. The data analysis protocol was adapted from earlier publications with no further modifications (Dufes et al., 2004).

(1) Flux(J)=(dM/dt)/A

And,

(2) Permeability(Papp)=Flux/Cd

*M* is the cumulative amount of drug transported to the receiver chamber.

*A* is the cross-sectional surface area of the inserts.

Cd is the donor concentrations at t = 0.

### 3.4. Cytotoxicity data analysis

Since the RFU is directly proportional to the number of dead cells, the percentage of viable cells was calculated by the following equation:

%ofViability=100−((AverageRFUoftestcompound−BackgroundRFU)/positivecontrolRFU.

## 4. Results and discussion

**Figure 1.**
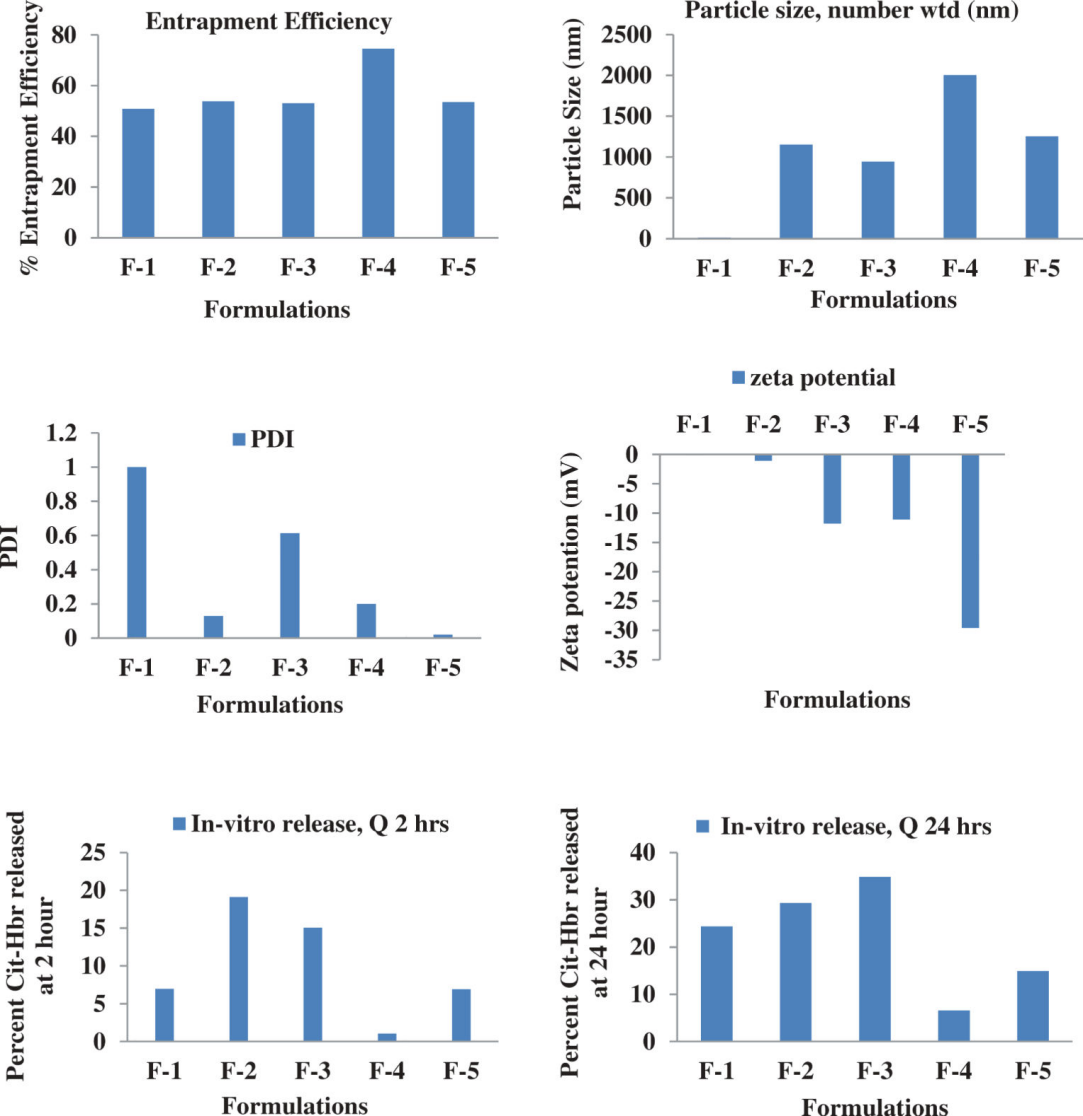
% of Entrapment Efficiency, number weighted particle size (nm), PDI, Zeta potential (s^−1^) and In-vitro percentage of drug release at 2 h and 24 h of the five liposomal formulations.

**Figure 2.**
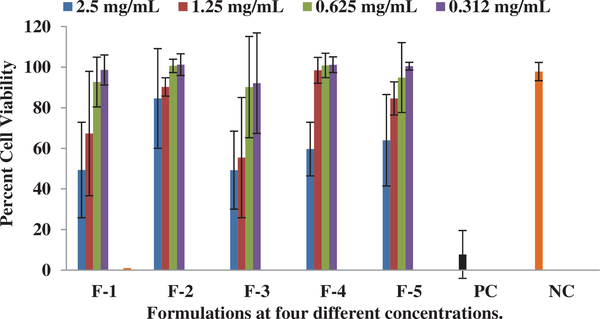
Represent the Percentage of RPBEMECs viability for the five formulations at 2.5, 1.25, 0.625 and 0.312 mg/mL, respectively. Each data point represents the mean ± SD of four determinations.

**Figure 3.**
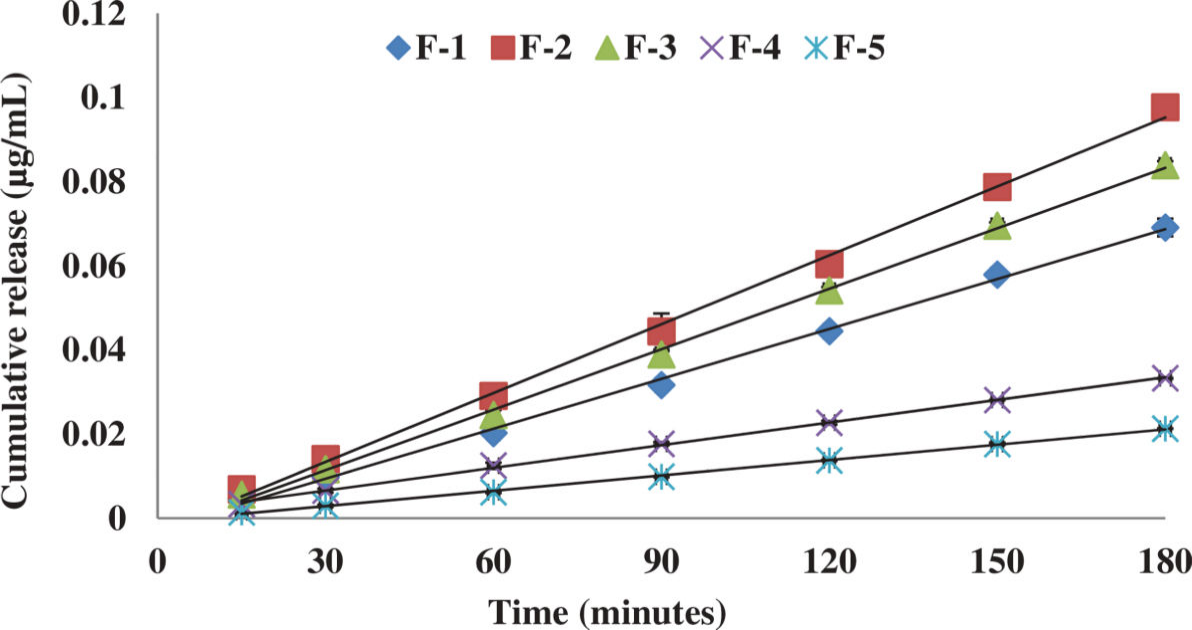
Unidirectional (A-B) transport study through the rat brain endothelial cells monolayer represents Papp (cm/min) of the five liposomal formulations (at 2.5 mg/ml conc.) from 15 min to 180 min. Each data point represents the mean ± SD of four determinations.

**Figure 4.**
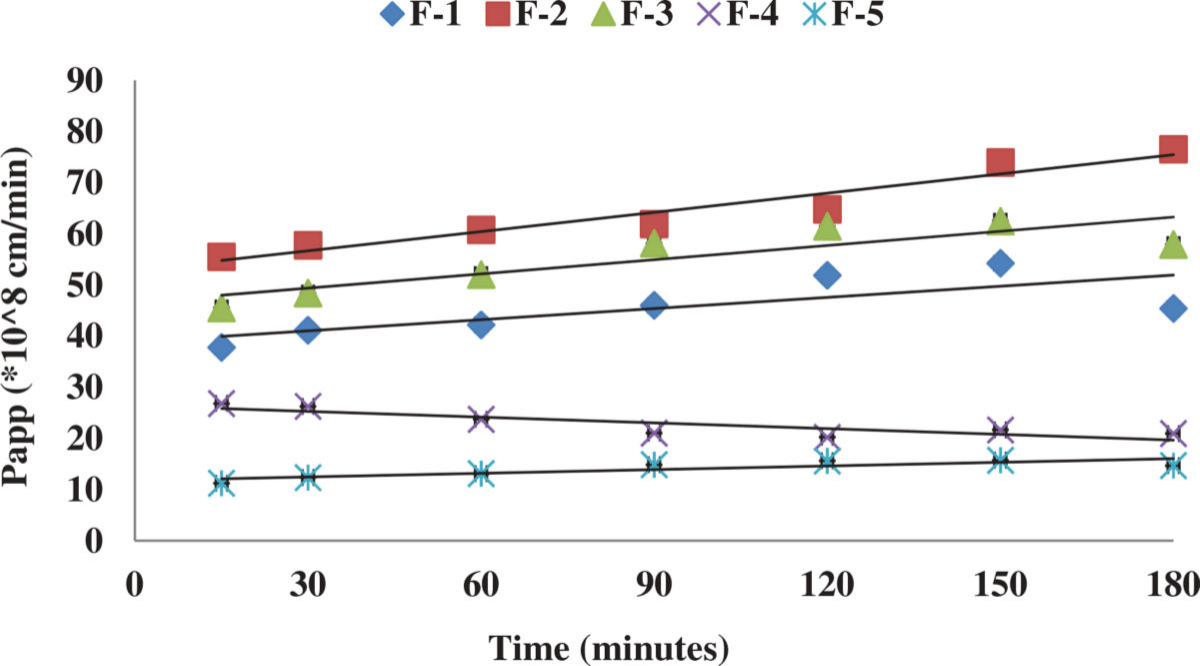
Unidirectional (A-B) transport study through the rat brain endothelial cells monolayer represents Papp (cm/min) of the five liposomal formulations (at 2.5 mg/ml conc.) from 15 min to 180 min. Each data point represents the mean ± SD of four determinations.

**Figure 5.**
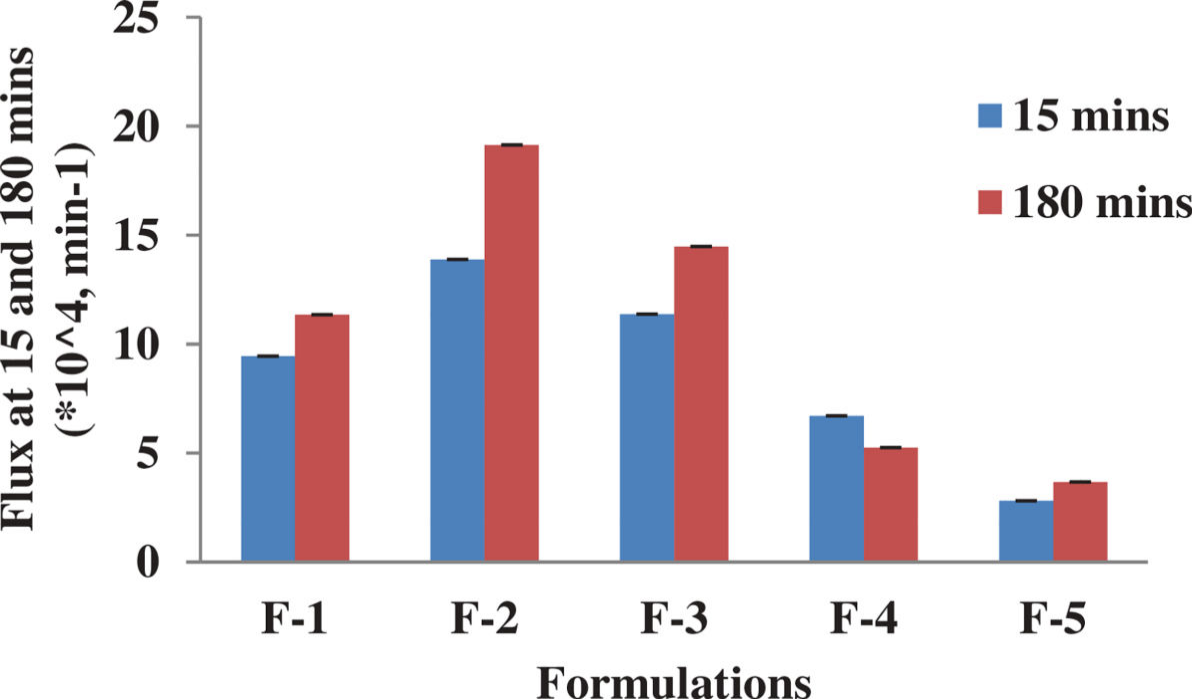
Unidirectional (A-B) flux (min^−1^) of the five liposomal formulations (2.5 mg/ml) at 15 and 180 min. Each data point represents the mean ± SD of four determinations.

In the current study, we have successfully evaluated an in-vitro rat brain endothelial cell model to evaluate the transport characteristics of five pegylated liposomal formulations of different physiological characteristics. RPBEC cell line utilized in the study was reported to demonstrate similar morphological characteristics of primary brain endothelial cells (Roux & Couraud, 2005). Our earlier published study reported that the physiological characteristics of Cit-Hbr liposomal formulations were significantly influenced by formulation additives (Kamal et al., 2015). The particle size, entrapment efficiency, PDI, surface charge and release parameters of the five formulations are shown in Figure 1. Formulation F-4 containing higher amount of phospholipid, cholesterol, diacetyl phosphate, PEG and drug load had better entrapment efficiency with larger particle size. The positive impact of these variables on entrapment efficiency and particle size can be attributed to higher phospholipids enabling the formation of more liposomal vesicles and larger internal volume for increased drug entrapment (Kamal et al., 2015; Xu, Khan, & Burgess, 2011). Further, higher amounts of cholesterol were predicted to reduce drug loss from liposomal vesicles (Kamal et al., 2015). The positive influence of the DCP on the entrapment efficiency can be due to interactions between the positively charged Cit-Hbr conjugate base and negatively charged DCP. The negative influence of NAG on entrapment efficiency can be due to repelling action of the charged drug molecule by positive ammonium molecule (Rengel et al., 2002). However, the effect of NAG on drug entrapment diminished with larger liposomal particle size enabling higher drug loading efficiency. F-1 formulations containing smaller amounts of phospholipid, DCP, cholesterol, PEG, NAG and drug amount demonstrated decreased particle size with lower entrapment efficiency. The zeta potential of F-5 formulation was higher compared to the other formulations and this observation can be attributed to an increase in hydration volume from 10 to 30 ml. In-vitro release of the five formulations by Float-A-Lyzer dialysis tubes (cellulose ester dialysis tube, 1 ml, molecular-cutoff 50kD, Spectrum Laboratories, Los Angeles, CA) demonstrated sustained release characteristics (Figure 1). Cytotoxicity data indicated cell viability (>90%) for all five formulations within a drug concentration range of 0.3–1.25 mg/ml (Figure 2). Cytotoxicity data suggested that the formulations were well tolerated by the cells up to a concentration of 1.25 mg/ml. TEER of the monolayer was ≥200 Ωcm2 and Lucifer Yellow (LY) transport was less than 1% before and after the transport study in RPBMECs (Table 2). TEER and Lucifer Yellow permeability values affirmed the maintenance of cell monolayer integrity during the drug transport study. Further, the TEER and LY values suggested that the RPBMECs monolayer model can be employed as a robust screening tool for future drug transport studies targeting GLUT transporter on BBB. The Cit-HBr transport studies from the apical to basolateral site are shown in Figures 3, 4 and 5. The commutative release and Apparent drug permeability (Papp) values for five formulations ranged from 0.001 to 0.1 μg/mL and 5.01 × 10^4^ to 15 × 10^4^ cm/min, respectively (Figure 3 and 4). The flux value ranged from 6.71 to 13.88 min^−1^ at 15 min and 3.67 to 19.14 min^−1^ at 180 min. As shown in Figures 3, 4 and 5, most of the Cit-HBr was transported across the RPBMECs by the formulation F-2 that achieved 53.86% of entrapment efficiency and exhibited particle size of 1153 nm, PDI of 0.13 and Zeta potential of −1.09.

## 5. Conclusions

**Figure 6.**
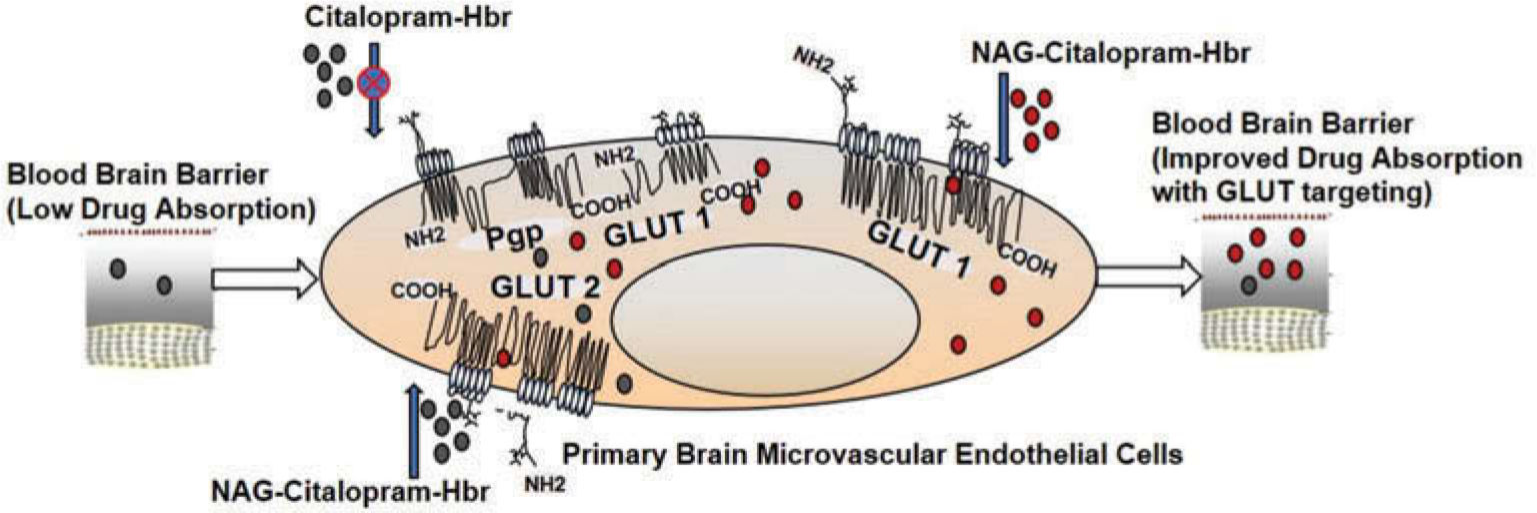
Schematic representation of GLUT transporter targeting of NAG-Citalopram-HBR on primary brain microvascular endothelial cells and improved absorption of the drug across BBB.

The study demonstrated successful targeting of GLUT transporter employing NAG coated pegylated liposomal formulation (Figure 6). Further, the study demonstrated that rat brain endothelial cells can be employed as an in-vitro model for GLUT transporter screening studies on the BBB. The study discussed the successful formulation and physio-chemical characterization of NAG-coated pegylated multilamellar liposomes with acceptable drug entrapment efficiency. The studies indicated promising cytotoxicity data with formulations being well tolerated for up to 1.25 mg/ml.

The Papp values provide initial proof that NAG-coated liposomal formulations can be explored as a promising drug transport vehicle for effective drug delivery across BBB.

## Figures and Tables

**Table 1.** Composition and processing variables of five liposomal Cit-HBr formulations

Batch	Phospholipon (mg)	Cholesterol (mg)	Dicetyl Phosphate^a^	PEG^a^	NAG^a^	Drug^b^ concentration	Hydration volume, ml
F- 1	200	100	6	10	12	5	10
F- 2	100	50	3	5	4	5	10
F- 3	200	100	6	10	4	5	10
F- 4	200	100	6	10	8	20	10
F- 5	200	100	6	10	8	10	30

a The amounts were added as percentages of the total lipid in the formulation.

b Drug concentration mg/mL in the hydration medium

**Table 2.** Percentage of the Lucifer Yellow leakage (n-4) before and after the drug transport study through the rat brain microvascular endothelial cells in the 12 inserts containing 24-well plate

**BEFORE**	**F-1**	**F-2**	**F-3**	**F-4**	**F-5**
A (×10^−4^)	5	4	5	4	4
B (×10^−4^)	3	4	5	4	3
C (×10^−4^)	5	4	4	7	4
D (×10^−4^)	4	4	4	4	4
Mean (×10^−4^)	4.25	4	4.5	4.75	3.75
StDev (×10^−5^)	±8.3	±0	±5.0	±13	±4.3
**AFTER**	**F-1**	**F-2**	**F-3**	**F-4**	**F-5**
A (×10^−4^)	8.3	4.4	13.5	6.1	6.4
B (×10^−4^)	6.8	5.2	12.5	17.7	6
C (×10^−4^)	6.2	3.4	7.9	9	5
D (×10^−4^)	5.8	6.7	14.7	20.9	5.4
Mean (×10^−4^)	6.7	4.93	12.15	13.43	5.7
StDev (×10^−5^)	±9.5	±12.0	±0.3	±6.0	±5.0
